# The importance of crash reporting requirements and how they affect analyses of factors associated with wildlife-vehicle collisions

**DOI:** 10.1371/journal.pone.0335517

**Published:** 2025-10-24

**Authors:** Amalie Victoria Jørgensen, Patricia C. Cramer, Wyatt M. Mack, Susan N. Ellis-Felege

**Affiliations:** 1 Department of Biology, University of North Dakota, Grand Forks, North Dakota, United States of America; 2 Wildlife Connectivity Institute, Gallatin Gateway, Montana, United States of America; 3 North Dakota Department of Transportation, Bismarck, North Dakota, United States of America; Tongji University, CHINA

## Abstract

Several factors have been identified to increase wildlife-vehicle collisions (WVCs). These may be species specific, ecological, temporal, driver related or related to road characteristics. To recommend effective mitigation strategies, it is imperative to understand the underlying factors driving WVCs. Our objective was to use crash data to identify factors that may contribute to reported WVCs in North Dakota and determine if changes in crash reporting affected the results. In 2013, crash reporting requirements were changed to only include crashes with human injuries or fatalities, and in 2019 increased the property damage threshold from $1,000 to $4,000. Based on reported crashes from North Dakota Department of Transportation (NDDOT), we compared results of the full crash dataset (2005–2022), pre-2013, post-2013, and fatalities and injuries data (2005–2022). We extracted factors from crash reports and hypothesized that different reporting causes different conclusions. We analyzed species, crash time, season, driver age and sex, and road type, speed limit, and lighting conditions. Deer were involved in >95% of the 30,476 reported wildlife-vehicle accidents. Annually reported WVCs averaged 3,488 in pre-2013 data, 133 in post-2013 data, and 40 in the fatalities and injuries dataset. We found significant differences in total number of WVCs with respect to species group, season, time of day, and road features (i.e., road type, lighting conditions, speed limit). The driver’s age was only significant in the fatalities and injuries dataset. Given the reduction in sample size after 2013, we did not detect statistically significant results in the post-2013 data for the effects of season. However, there were statistically significant differences in all-years, pre-2013, and fatalities and injuries datasets with respect to season. This suggests the ability to detect important factors is influenced by reduced sample size from reporting requirement changes in 2013 that limited our ability to make interferences on factors that affect WVCs in North Dakota.

## Introduction

Wildlife-vehicle collisions (WVCs) are a major public safety issue and pose challenges to wildlife conservation. To propose the most effective mitigation, it is important to understand contributing factors to WVCs. In the United States alone, based on reported crashes, WVCs account for more than 200 human deaths, over 26,000 human injuries, and over $10 billion in societal costs [1–3] Nationwide, WVCs account for approximately 5% of all collisions [3]. With the expectation of several million kilometers of new roads to be built globally [4], the number of WVCs are also expected to increase [5]. In addition, approximately 365 million vertebrates are killed in vehicle collisions annually in the United States [5,6]. More importantly, roughly one to two million large mammals are hit by vehicles each year in the United States [7], causing the most property damage and human injuries of all animal groups [8,9]. Furthermore, roads cause habitat loss and fragmentation and, in some cases, barriers between wildlife populations [10]. This ultimately inhibits habitat connectivity and forces wildlife to cross busy roadways [6,11].

There are several ways to reduce WVCs and different mitigation methods have been tested and developed over the years [12]. The main goal of these efforts is to keep wildlife off roadways or from becoming involved in WVCs by either altering human behavior, animal behavior, or both [12]. Understanding the factors related to WVCs is important in prescribing the most effective mitigation types and locations. Several factors have been identified that increase WVCs. Studies have found that roads bisecting or adjacent to wildlife habitat that provide food, water, and shelter for wildlife generally experience more WVCs [13]. In addition, roadways surrounded by agriculture have been found to increase the chances of deer-vehicle collisions [14]. When it comes to road and traffic characteristics, factors such as increased traffic volume, higher speed limits, wider roads, low visibility, and riparian corridors are all positively correlated with increased WVCs [13,15]. As a result, understanding some of the underlying factors that cause an increase in WVCs in a specific geographic area is important in order to select the appropriate mitigation techniques.

WVC research is most often based on crash data from law enforcement officer reports or carcass data from ecological surveys [16]. Some researchers also collect data through citizen science [17] or through LIDAR (Light Detection and Ranging) and camera scanners in vehicles [18]. However, reported crashes rarely show the total number of WVCs because not every accident is reported [19,20]. Despite this, crash reports are one of the primary sources of information for WVCs because they are often the data most consistently collected. However, reporting requirements often vary among states and across countries leading to inconsistencies in these crash data sources. These varied requirements then may result in a biased perspective of the types of WVCs that occur, the species involved, and characteristics of crash locations, or other factors that might provide valuable insights for mitigation efforts and facilitating wildlife connectivity efforts. Nonetheless, even though the crash data may be limited, it has valuable information for exploring characteristics that may have led to the collisions.

North Dakota is a rural U.S. state with over 18% of all reported crashes attributed to wildlife [3]. Prior to July 2013, the state reported all accidents with property damage at or above $1,000 including crashes with wild and domestic animals. During this period, collisions averaged in the thousands annually and cost North Dakota citizens millions of dollars [3]. In 2013, the reporting requirements changed and only collisions that include human injury or death were reported (63^rd^ Legislative Assembly of North Dakota House Bill 1123). Furthermore, in 2019, the reported property damage threshold for reporting was increased from $1,000 to $4,000 (66^th^ Legislative Assembly of North Dakota House Bill 1065); however, this did not cause an overall dramatic shift in the number of reported collisions compared to the 2013 changes. There is a need to better understand how the reporting changes might affect data available for analyses and to make mitigation decisions.

Our objectives were to analyze the influence of factors available in crash reports on North Dakota’s state and federal roads on WVC and to determine if commonly documented crash factors that affect WVCs in other regions and states are important in North Dakota. Specifically, we wanted to evaluate the influence of the reported species groups, temporal characteristics, driver demographics, and road characteristics that were associated with reported WVCs in North Dakota based on what has been identified as important in the literature and how they may affect the number of WVCs. More importantly, we were interested in characterizing if these factors varied across two time periods of different crash reporting (pre- and post-2013) in addition to the entire 18-year dataset (2005–2022) to better understand how reporting effort influences inferences used to make mitigation decisions for WVCs in the state and region. Lastly, we wanted to determine if there was a difference between the time periods and the WVCs resulting in human fatalities and injuries (2005–2022).

### Hypotheses

We expected the highest proportion of crashes to occur during fall when breeding and hunting season are occurring for large mammals, specifically deer [21]. Additionally, a higher number of WVCs are expected during dusk and dawn because peak animal movement happens during these hours and lighting conditions are less favorable for easy detection by drivers [22,23].

We were interested in understanding the role of driver demographics in WVCs. Research demonstrated that male drivers are more frequently involved in WVCs than female drivers [24,25]. Therefore, we expected to see a higher number of WVCs involving male drivers. North Dakota accident reports also include if a young driver was involved in the crash. Burton et al. [26] found that drivers less than 18 years of age are involved in less WVCs compared to other ages, and we therefore hypothesized that we would see the same trends in North Dakota’s wildlife crashes.

Road and traffic characteristics can be important factors associated with crashes. Studies have found that roads with higher speed limits are positively correlated with an increase in moose- and deer-vehicle collisions [15,27,28]. However, researchers have found that speed limits would have to be reduced to 45 mph (72.4 kph) or less to decrease the number of WVCs occurring [15]. Given the greater stopping distance of vehicles traveling at faster speeds, we expected to see more collisions occurring within these higher speed limits. We expected road type may affect WVCs. Larger roads with more lanes and divided medians may be less likely to have crashes due to the barriers created by high levels of daily traffic; wildlife are known to reduce attempts to cross roads when traffic volumes reach a certain point, depending on the species [29–31]. Research has documented that wildlife have a tendency to avoid crossing roads where annual average daily traffic (AADT) is 10,000 or higher [29,31]. We also hypothesized that more WVCs would occur on unlit roads (no streetlights). With less light, it is harder to notice animals approaching and crossing the roadways, giving the driver less time to react and stop [32].

Lastly, we hypothesized that reporting methods matter. We expected that the change in reporting requirements in 2013 and the reduced sample size would result in an increased variability of the data across the all-years dataset, leading to a reduced ability to detect differences in factors affecting WVCs.

## Methods

We used reported crash data collected by the North Dakota law enforcement officers from 2005–2022. In North Dakota during this period, there were legislative changes made in 2013 and 2019 as noted above. Since the largest change was made in 2013, we examined data as pre-2013 (2005–2012), post-2013 (2014–2022), and all-years (combined dataset) across each factor of interest to determine the role reporting played in our interpretations of the analysis. This division in years resulted in the pre-2013 dataset consisting of eight years of data and nine years of data in the post-2013 dataset. Additionally, we decided to extract data on crashes only including fatalities and injuries (2005–2022). We chose to add this final dataset because these accidents are the majority of the ones reported post-2013.

The collected data was reported in one of three categories when crashes with wild animals occur; deer, small animal, or other large game. In the attribute table of the GIS shapefiles, each accident report explicitly included the following information: date, time, location, species group, lighting conditions (e.g., dark (with or without street lights), dusk, and dawn), road characteristics (e.g., divided and non-divided roads), driver demographics (e.g., sex and age), among other details (e.g., number of vehicles involved, etc...). We summarized the data and displayed it graphically with specific interest in species, crash hour, crash day, driver demographics, season, roadway characteristics, lighting conditions, and speed limits, as these attributes are some of the most common identified in literature as contributing factors to WVCs [33–36].

Under “first harmful event,” species were reported as deer (while not often speciated this included mule deer (*Odocoileus hemionus*) and white-tailed deer (*Odocoileus virginianus*)), small animals (e.g., raccoon (*Procyon lotor*) and striped skunks (*Mephitis mephitis*)), or other large game (ungulates, e.g., elk (*Cervus canadensis*), moose (*Alces alces*), bighorn sheep (*Ovis canadensis*), and pronghorn (*Antilocapra americana*)). Both mule deer and white-tailed deer are found in North Dakota. However, these two species are not usually identified to species level during crash reports, so we are unable to determine the exact proportion of each deer species involved in WVCs. It is worth noting that white-tailed deer are found statewide, whereas mule deer are typically more restricted to the western part of the state with the largest populations west of the Missouri River [37].

Time of day was recorded using military time and ran from 00:00–23:59. We explored all days of the week (Monday through Sunday) to evaluate temporal shifts associated with the work week and weekends. For driver sex, the data was divided into male and female, and for age we examined two groups: ≤ 19 years old and over 19 years old. This age cut-off was chosen because North Dakota accident reports include a column for a young driver (i.e., ≤ 19 years old) and research suggests that young drivers may be involved in less WVCs when compared to their proportion of the population and other age groups [26]. The seasons were divided into winter (December-February), spring (March-May), summer (June-August), and fall (September-November). Roadway characteristics were categorized as divided, not divided, and one way. Lighting conditions were grouped as dark (road lighted), dark (road unlighted), dawn, daylight, and dusk. Road lighted and road unlighted refers to the presence and absence of streetlights. The posted speed limits reported in the crash data range from 0 to 80 miles per hour (mph) (0 to 128.7 kilometers per hour, kph). While no posted speed limit in North Dakota currently exceeds 75 mph, there were crash reports with the speed limit of up to 80 mph. Because this is the speed recorded in the crash report, this is likely due to drivers speeding. We chose to retain the original data, recognizing this may also have been an error in the record, but it was most likely reflective of one of the higher posted speed limits. Further, we divided these into three groups: ≤ 45, 45–65, and ≥65 mph. This was done to better incorporate the expected values based on the posted speed limits across the state.

We performed a one-way ANOVA (Analysis of Variance) for the factors (or treatments) of weekdays, season, road type, and lighting conditions by analyzing the total number of WVCs in each year for each of the respective treatments. One-way ANOVAs were chosen because we compared the means of three or more groups within each factor. For each factor, we conducted four ANOVA evaluations. One on the all-years dataset (18 years of data), one on the pre-2013 (8 years), one on the post-2013 (9 years of data), and one of the fatalities and injuries (18 years of data). We were also interested in examining if season and crash hour were associated with each other. To do this, we performed a two-way ANOVA to be able to account for the two factors (season and time) of interest and their interaction. When differences were detected from the ANOVA, a Tukey test was conducted to discern specific differences. To examine if there were differences in the distribution of driver demographics of age (over and ≤19) and sex, we conducted a Chi-square analyses. The expected value for age was based on reports from Statistical Atlas which shows that 6.58% of North Dakota’s residents fall within the ages 15–19 years old [38]. Expected value for driver sex was based on reports from the Federal Highway Administration which states that men make up 62% of all drivers [39]. Speed limits were categorized into three groups (i.e., ≤ 45, 45–65, and ≥65) and analyzed using a Chi-square where the expected values for the proportion of roads in each group based on data shared by the NDDOT. Similar to the ANOVAs, four chi-square analyses were conducted for each factor (all-years, pre-2013, post-2013, and fatalities and injuries). For all tests, statistically significant differences were determined to be present when P < 0.05. All analyses were conducted in RStudio (Version 4.3.0) using the packages ggplot2, dplyr, ggpubr, tidyverse, and car.

## Results

After removing datapoints outside of North Dakota, the crash data included 30,476 WVC accidents reported between 2005 and 2022. The majority (91.6%) of the collisions were reported pre-2013. This resulted in an annual average of 3,488 reported WVCs in pre-213 compared to only 133 reported accidents post-2013 (Table 1). When examining the fatalities and injuries dataset, 727 accidents were reported between 2005 and 2022. This resulted in an average of 40 reported WVCs annually.

**Table 1:** Summary statistics including total number of crashes reported, the annual average, and standard deviation among the four datasets used.

	All-years 2005 - 2022	Pre-2013* 2005-2012	Post-2013* 2014-2022	Injuries and Fatalities 2005-2022
**Total WVCs**	30,476	27,904 (~92%)	1197 (~4%)	727
**Annual average**	1792	3488	133	40
**Standard Deviation**	1690.7	340.4	23.9	10.3

*Note that we omitted 2013 crashes since reporting changed midway through the year.

### Species involved

**Fig 1:**
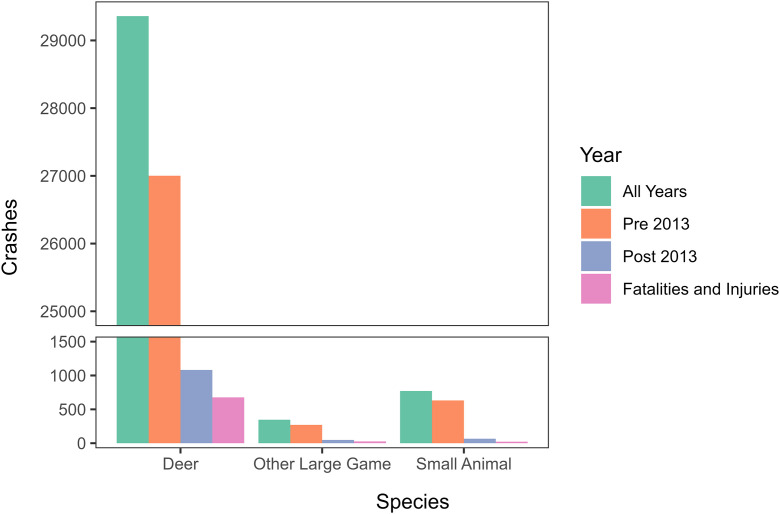
Reported wildlife-vehicle collisions in North Dakota shown by the total number and type of wildlife occurring in North Dakota grouped across all study years (2005-2022), pre-2013, post-2013, and fatalities and injuries (2005-2022) reported accidents to capture the changes in reporting requirements. Note the axis break to best demonstrate the magnitude of difference in the deer numbers compared to other species groups.

As we expected, deer were involved in 96.3% (91.9% reported pre-2013) of all WVCs in North Dakota (Fig 1). Small animals accounted for 2.5% of the reported WVCs, with almost 82% of these being reported pre-2013. The remaining WVCs (1.2%) were reported as other large game (approximately 78% of these were reported pre-2013). Similar to the previous research, over 90% of the WVCs reported as fatalities and injuries were deer-vehicle collisions. None of the species groups include details about specific species hit.

### Temporal characteristics

#### Days of the week.

Regardless of which of the four datasets were analyzed, we detected no statistical difference in the number of WVCs occurring during the different days of the week (Table 2). While not statistically significant, there was a trend for higher numbers of WVCs to occur on Thursdays, Fridays, and Saturdays.

**Table 2:** Statistical results summarized to show the test statistic, degrees of freedom and the p-values from each factor based on reported wildlife-vehicle collisions in North Dakota.

	All Data (18 years)	Pre (8 years)	Post (9 years)	Fatalities and Injuries (18 years)
**Temporal Factors**				
Day of the Week	F_6,119_ = 0.06, p = 0.99	F_6,49_ = 2.25, p = 0.054	F_6, 56_ = 1.02, p = 0.42	F_6,119_ = 0.89, p = 0.507
Season	F_3,68_ = 2.83, p = 0.05	F_3,28_ = 88.55, p < 0.001	F_3,32_ = 2.41, p = 0.09	F_3,68_ = 27.3, p < 0.001
Season:Time	F_9,170 _= 1.4, p = 0.19	F_9,70 _= 34.4, p < 0.001	F_9, 80_ = 8.2, p < 0.001	F_9,170_ = 10.57, p < 0.001
**Driver Demographics**				
Age	X^2^_1, 30476 _= 2.29, p = 0.13	X^2^_1, 27904 _= 1.99, p = 0.16	X^2^_1, 1197_ = 2.34, p = 0.12	X^2^_1,727_ = 4.47, p = 0.034
Sex	X^2^_1, 30476 _= 0.65, p = 0.42	X^2^_1, 27904_ = 4.84e-05, p = 0.99	X^2^_1, 1197 _= 2.32, p = 0.13	X^2^_1, 727 _= 0.18, p = 0.67
**Road Conditions**				
Road Type	F_3,68_ = 12.58, p < 0.001	F_3,28_ = 698, p < 0.001	F_3,32_ = 180.9, p < 0.001	F_3,68_ = 85.52, p < 0.001
Lighting Conditions	F_4,85_ = 11.3, p < 0.001	F_4,35_ = 326.8, p < 0.001	F_4,40_ = 131, p < 0.001	F_4,85_ = 70.34, p < 0.001
Speed Limits	X^2^_2, 30476_ = 72.6, p < 0.001	X^2^_2, 27902_ = 74.5, p < 0.001	X^2^_2, 1197_ = 38.7, p < 0.001	X^2^_2,727_ = 107.48, p < 0.001

ANOVAs were run on days of the week, season, interaction of season and time (season:time), road type, and lighting conditions. Chi-squares were run on age, sex, and speed limit. Statistical significance was p < 0.05.

#### Seasons.

Based on total numbers of North Dakota’s WVCs being visualized in a graph, the species group small animal is evenly distributed throughout the seasons. The group other large game has a peak during the fall. Additionally, based on reported collisions graphed by season, we observed deer crashes were highest in the fall, followed by summer (11,655 and 6,998 reported deer crashes, respectively).

**Fig 2:**
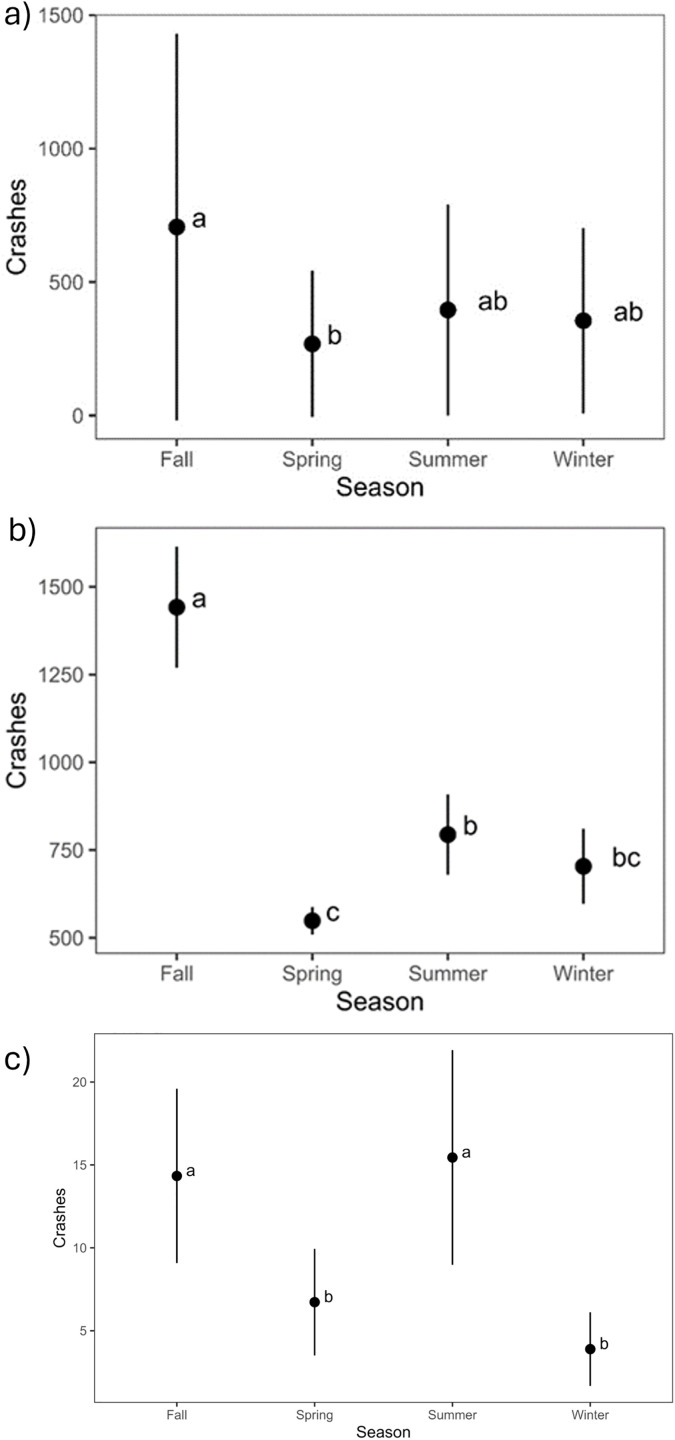
Mean number (± 95% confidence intervals) of annual wildlife-vehicle collision across seasons in North Dakota during the (a) all-years (2005-2022), the (b) pre-2013 survey period (2005-2012), and (c) the fatalities and injuries data showing a greater number of crashes occur in the fall (September through November) season. Additionally, the fatalities and injuries dataset show no difference between fall and summer. Means with different letters (e.g., a, b, and ab) were significantly different via a Tukey test.

We found a significant season effect within the all-years dataset, the pre-2013 dataset, and the fatalities and injuries dataset (p**-value* *< 0.05; Table 2). No significant difference in season was detected in the post-2013 dataset. A *post hoc* Tukey test was conducted on these datasets and fall had a significantly higher number of WVCs than the other three seasons in the all-years and pre-2013 datasets (Fig 2). In the all-years dataset, spring was significantly different from summer and winter as well (Fig 2a). Further, in the pre-2013 dataset, summer was found to have significantly more WVCs than spring (Fig 2b). Lastly, in the fatalities and injuries dataset, there was no significant difference between fall and summer, but the two seasons had significantly higher WVCs than spring and winter (Fig 2c).

#### Time of day.

More WVC accidents occurred between 06:00 and 07:00 in the morning and between 20:00 and 22:00 at night. From the two-way ANOVA, we found no significant difference in the season:time interaction for the all-years dataset. There was, however, a significant difference in season:time in both the pre- and post-2013 datasets (Table 2). Our results also showed a significant difference in season:time in the fatalities and injuries dataset. These results suggest that there is a connection between season and time of day. More specifically, this shows that season and time of day has an impact on when WVCs occur in that there is variation in dusk and dawn times in North Dakota across the seasons. For example, in winter dusk occurs near rush hour (e.g., 5 pm) compared to summer when it is much later (e.g., 9 pm or later).

### Driver demographics

#### Age.

Only 9.4% of the drivers involved in wildlife-vehicle accidents in North Dakota were considered young drivers (≤19 years old). Of North Dakota’s residents, 6.48% are considered to be within the age range of 15–19 years old [38]. When compared to the expected distribution of drivers in North Dakota, no significant difference was found in the all-years, pre-2013, and post-2013 data. However, for the fatalities and injuries dataset, a significant difference between the proportion of each age class of drivers involved in WVCs was different than the expected proportion of North Dakota drivers [38] in each age class (p = 0.034; Table 2).

#### Sex.

Approximately 60% of drivers involved in WVC accidents in North Dakota were male drivers. Nationwide, approximately 62% of drivers are male [39]. The Chi-square analyses revealed no statistically significant result for driver sex when compared to the national average of the proportion of male drivers in the population (Table 2).

### Driving conditions

#### Speed limits.

**Fig 3:**
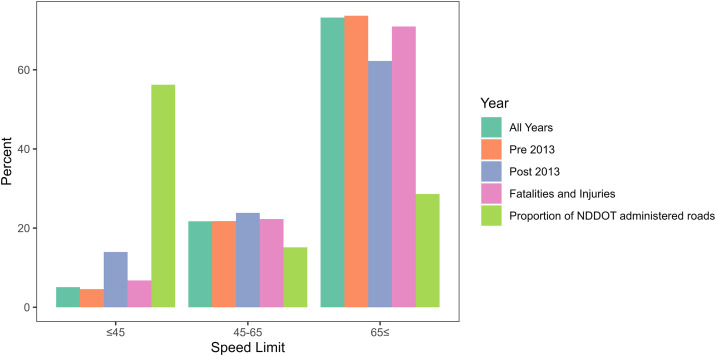
The percentage of reported wildlife-vehicle collisions (WVC) that occurred within three different speed limit categories. The results are presented based on four different WVC datasets (all-years, pre-2013, post-2013, and fatalities and injuries) and compared to the actual proportion of North Dakota state highway roads within each posted speed limit category (Proportion of NDDOT administered roads).

While only approximately 25% of North Dakota’s roads have speed limits greater than 65 mph (104.6 kph), the majority of reported WVCs (73.2%) occurred on these roads. The second highest (21.7%) occurrence of WVCs occurred on 45–65 mph (72.4–104.6 kph) roads. After running Chi-square on the speed limit groups (≤45, 45–65, and 65≤), we found that there was a significant difference between the groups in all four datasets (Table 2). In general, we found a higher proportion of crashes were at higher speed limits (greater than 65 mph) than the proportion of available roads and lower a proportion of crashes at the lowest speed limits (less than 45 mph) than we expected (Fig 3).

#### Road type.

**Fig 4:**
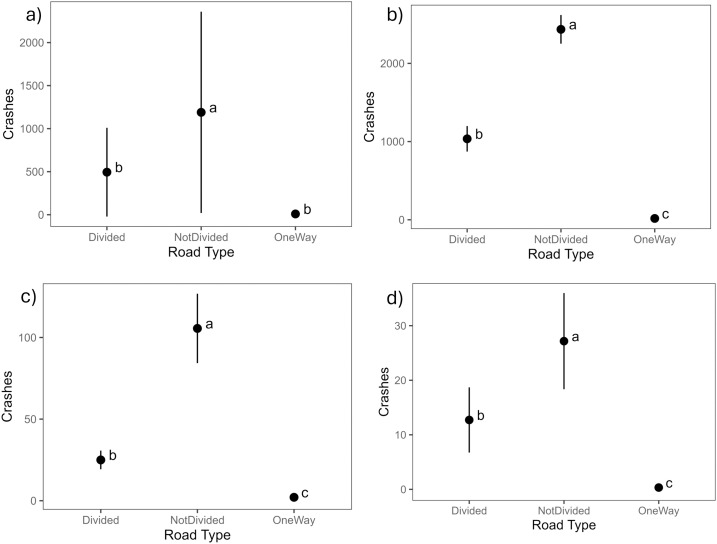
Tukey test results based on road type from reported wildlife-vehicle collisions in North Dakota. The results show that significantly more accidents occur on non-divided roadways. The graphs show results from (a) all-years dataset, (b) pre-2013 dataset, (c) post-2013, (d) fatalities and injuries dataset.

Approximately 70% of the reported WVCs between 2005 and 2022 occurred on non-divided roadways, and the analyses confirmed that significantly more WVC accidents were reported on non-divided roadways (two-lane highways) in North Dakota (Fig 4). Additionally, significantly more accidents occurred on divided roads compared to one-way roadways (Fig 4b, c, and d).

#### Light conditions.

**Fig 5:**
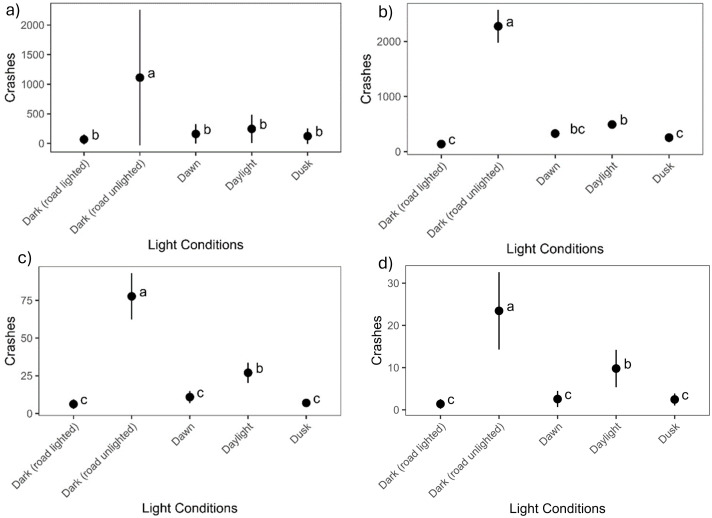
Light condition Tukey test results showing that there are significantly more wildlife-vehicle collisions occurring on dark, unlit roads in North Dakota. The results include the mean number and its 95% confidence intervals. The three graphs show the results based on (a) all-years, (b) pre-2013, (c) post-2013, (d) fatalities and injuries datasets.

After graphing the reported crashes relative to light conditions, we observed that the majority of the crashes fell within the “dark (road unlighted)” category. Through ANOVAs, we found significant differences in all four-year groupings of crash datasets (p < 0.001 in all four datasets) for light conditions. The *post hoc* Tukey test showed that dark, unlit roads had a higher number of WVCs than all other conditions across all three datasets (Fig 5).

### Implications of report requirements

Analyzed factors were similar among the all-years, the pre-2013, post-2013, and the fatalities and injuries datasets, including broad error bars (95% confidence intervals) for the all-years data compared to the other three. These results are likely seen due to the difference in reporting and therefore the different sample size. Thus, analyzing the all-years dataset without considering the reporting change, may make the final results difficult to interpret.

## Discussion

North Dakota has one of the highest proportions of wildlife-vehicle collisions in the country (18%) [3], and our objective was to determine if the factors commonly identified across the U.S. were contributing to WVCs in the state as well. Using reported crash data from 2005–2022, we found that factors associated with WVCs in North Dakota are consistent with the factors identified in literature (i.e., season, species) for WVCs across the country.

Specifically, we found deer to be the species group most involved in WVCs in North Dakota, which was not surprising since deer is one of the most abundant and widely distributed animals across North America [37]. Given that deer were the most commonly reported animal to be involved in WVCs, we expected an increase in deer crashes during the fall due to breeding activity and hunting pressure as observed in other studies [21,22]. Our hypothesis was supported in the all-years and the pre-2013 data; however, we did not find a seasonal difference in the post-2013 dataset. We suspect that current crash reporting methods are providing insufficient data for detecting this difference since we found a difference in the years with high sample sizes. Where differences existed, we found fall higher than other seasons and summer higher than spring which has been observed in other studies [22,40]. In South Dakota, a rural state with many similarities to North Dakota, research also found the fall season to have the highest deer-vehicle collisions [41]. This second peak may be the result of increased activity by female deer as they move to fawning grounds and when the yearlings are turned away by their mothers and begin independent, inexperienced lives [40]. Additional reasons for increased activity this time of the year may include vegetation green up from road run-off and mineral deposits from de-icing agents may be attracting wildlife such as deer to roads in the spring [42].

We found significantly more accidents occurred on non-divided roadways than occur in the state. These non-divided roadways are typically two-lane highways. Additionally, we saw that during the pre- and post-2013 datasets, there were significantly more accidents that occurred on divided roads compared to one-way roads. Divided highways typically have higher traffic volume which research has shown both discourages wildlife’s attempt to cross the roads and may have resulted in extirpation of the local populations along these roads; thus, these roads have lower WVCs [29,31]. Further, these results should be considered carefully, as North Dakota likely has more non-divided roads than those with medians. As a result, there are some limitations to analyzing this factor using an ANOVA. Based on the available data we obtained from only crash data, we did see a significant difference in road type; however, we suggest additional analysis using a Chi-square to explore the proportion of crashes relative to the proportion of roads of each type in the state.

Most North Dakota crashes occurred during dark conditions and most often on unlighted roadways (absence of streetlights). We did find a higher number of recorded WVCs during daylight than dusk and dawn. We expected dusk and dawn to have the highest crash rates due to peak animal movement that may also correspond to the morning and evening commute during these times in the fall, but found that most North Dakota WVCs occur during the dark, on unlit roadways followed by daylight. Research has found that streetlights may reduce WVCs up to 68% in an area, however, it is not clear if this is due to enhanced driver visibility or due to animals avoiding lighted areas [43]. Additionally, some animals may be attracted to artificial light, potentially causing more WVCs to occur [43]. Daylight also may have resulted in slightly more crashes than dusk and dawn since those periods likely have higher traffic volumes throughout the year. However, it should be mentioned that there are more unlit roadways than lighted roads in North Dakota, which causes some limitations to the ANVOA approach used. Again, the results are based on the data we had available, but the proportions of lit versus unlit roadways should be an area of future examination.

Previous research suggest male drivers are more commonly involved in WVCs [24,25]. Our reported data agrees that a higher number of WVCs involve male drivers in North Dakota; however, no significant difference was found when compared to the proportion of male drivers in the state suggesting there is no deviation from the distribution in drivers. Our findings of higher total counts of WVCs by male drivers are likely because there are simply more male residents in North Dakota [38] and more male drivers in general [44]. Our analysis found just over 9% of the drivers involved in North Dakota WVCs are considered young drivers (19 years old and younger), but we did not find a disproportionate number of accidents attributed to younger or older drivers than expected in the all-years, pre- and post-2013 datasets. In the fatalities and injuries dataset, the Chi-Square analysis found that North Dakota crash data had significantly more young drivers than expected (p = 0.034). This result is similar to other studies who found that younger drivers involved in WVCs were more likely to be injured [45,46]. This could be a result of younger drivers being more inexperienced and therefore they might have a slower reaction time. Overall, this differs from Burton et al. [26] who found that drivers younger than 18 years of age are less frequently involved. Even though our results showed few significant differences, it is important that future research makes sure to account for the proportion of expected versus observed values. If this is not done, it may appear like some factors are more important than they are.

A commonly proposed mitigation method is to restrict speeds to reduce WVCs. When looking at total numbers, most of the North Dakota WVCs were found to occur on roads with a 65-mph speed limit, followed by roads with a 55-mph speed limit. This is likely due to the increasing stopping distance for vehicles at higher speed limits. Additionally, at a higher speed limit, the driver has less time to react to potential wildlife entering the roadway [47]. After running the Chi-square analyses on the three speed limit groups in all three datasets, we found significant differences among all of them with respect to their proportions on the landscape (Table 2). Note that the reported crash datasets of North Dakota WVCs do not include other smaller, rural roads under other jurisdictions (e.g., townships) that may have reduced speed limits, and that wildlife may not perceive as barriers to movement due to low traffic volume. In order to influence WVCs on major highways, reduction in speed would be required to be 45 mph (72.4 kph) or less [15]. Furthermore, North Dakota is a primarily rural state where most of these roadways are unlighted, making it difficult to see potential wildlife on the road during the night and early morning, increasing the chances of WVCs [48]. Low lighting conditions coupled with higher speed limits likely increased the chances of WVCs.

We found that factors most strongly affecting WVCs differed among the datasets (e.g., significant difference between seasons in the pre-2013 and fatalities and injuries datasets, but not post-2013 or all-years). This is a concern as the change in North Dakota WVC crash reporting requirements did affect how crash data is interpreted and subsequently will impact mitigation plans to reduce these crashes. Moreover, with the reporting changes, the number of reported annual crashes was dramatically reduced, resulting in substantially different numbers of reported WVCs annually when comparing across longer temporal periods such as the 18 years we examined. Such changes increased variability around estimates, suggesting the all-years datasets that lumped reporting changes into them should not be used for decision-making associated with WVC mitigation. Evaluating the data by reporting method periods (pre-and post-changes or fatalities and injuries only) provides more accurate results, if sufficient data is available. Pre-2013, thousands of collisions were reported annually, resulting in a larger sample size available for evaluation of factors influencing WVC. Post-2013, there are, on average, less than 150 wildlife-vehicle crashes reported each year. The fatalities and injuries dataset was sparse, however, exploring this data allows for better understanding of where the most severe injuries occurred. This sparse data likely lacks the power to detect statistically significant results on factors that affect WVCs and biases the data towards more damaging crashes likely caused by larger animals (e.g., deer). If mitigation is focused on just safety, this provides a useful dataset for examining injury-prone areas; however, if it is focused on wildlife population connectivity and overall wildlife-crash reductions, other resources should augment the dataset for mitigation planning.

Studies that examine double samples where both carcass and crash data have been collected find that there are more carcasses than reported crashes [49,50]. Including collection of carcass data may provide increased sample sizes for WVC data analysis that facilitates more informed mitigation decision making. Carcass data could be collected while roadkill is being removed from roadways [51], using citizen science where the public can report roadkill [52], or working with state and federal agencies (e.g., Department of Transportations, Wildlife Agencies, US Postal Service carriers). Increasing interest has been placed on using the public through citizen science to report carcass data through a smartphone application that could augment current data from crashes [20], but it would require filtering to be sure multiple reports of the same carcass are not double counted. Carcass data collection assists transportation and wildlife agencies in further developing mitigation strategies to improve human roadway safety and improve wildlife connectivity.

A limitation of the study is that since more than 90% of all reported WVCs in North Dakota (and likely a high proportion of most states) are involving deer [45]. As a result, this study does not capture the breadth of all WVCs that could occur and that the factors associated with these collisions are heavily dependent upon the ecology of deer (e.g., peak times of movement during the day or time of year). Crash data would likely not have much information to assist in conservation efforts for small species that do not cause property damage or result in serious crashes that injury motorists.

While the focus of our study was to examine common factors impacting WVCs and the impact of reporting on interpreting the importance of those factors, we did not do an exhaustive examination of other characteristics that are associated with WVCs. This could include but is not limited to more detailed road characteristics (e.g., straight or curved roadways; [15]), traffic volume and characteristics associated with high human population centers [20], and vehicle type (e.g., passenger cars, trucks, motorcycles; [20]), and weather conditions (e.g., road surface conditions including snow, ice, rain; [15]). Ecological factors such as wildlife population trends, wildlife habitat and migration areas, vegetation near the road and in the right of way, land use such as agriculture, ecosystem type, human development of areas near the road, riparian areas, and other ecosystem factors are also found to be important [16,42,53,54], but were not examined here as our analyses were purely based on information extracted from the reported crash data available in North Dakota.

In conclusion, our analyses of crash data suggest that several of the primary factors (i.e., species, season, road features) that influence WVCs in other U.S. states align with factors in North Dakota WVCs [45]. However, the ability to detect these important factors is heavily influenced by the sample sizes and parameters associated with the collection of available crash data. For the state of North Dakota, the reporting rate requirements changed in 2013 reduces the amount of data available through crash reports. Sparse data (like the post-2013 dataset or the fatality/injury dataset) limits statistical power to detect differences that can be used to identify factors that are important in mitigation strategies that would reduce WVCs [55]. As a result, there may be missed opportunities to identify mitigation strategies that could reduce WVCs if a specific factor is not identified as important. If reporting approaches are not reverted back to increase sample sizes, data augmentation from other sources (e.g., carcasses, GIS analyses of land use and cover, human population, etc.), could provide improved information for mitigation efforts that enhance public safety and safe corridors for connecting wildlife populations. Moving forward, we suggest combining crash and carcass data to improve the analyses of WVC data. Additionally, if states change reporting approaches like North Dakota, they should be aware that changes in the amount of available crash data can result in different interpretations of where mitigation should be focused. With a better understanding of the common factors that increase the chances of WVCs, DOTs can better determine if, which, and where mitigation measures should be installed.
